# The use of mobile phone functionalities by patients with asthma and their desire to use for self-care purposes

**DOI:** 10.1186/s12911-020-01301-z

**Published:** 2020-10-30

**Authors:** Ehsan Nabovati, Mehrdad Farzandipour, Marzieh Heidarzadeh Arani, Hossein Akbari, Reihane Sharif, Shima Anvari

**Affiliations:** 1 Health Information Management Research Center, Department of Health Information Management and Technology, School of Allied Health Professions, Kashan University of Medical Sciences, Pezeshk Blvd, 5th of Qotbe Ravandi Blvd – Pardis Daneshgah, Kashan, Iran; 2 Pediatric Department, School of Medicine, Kashan University of Medical Sciences, Pezeshk Blvd, 5th of Qotbe Ravandi Blvd – Pardis Daneshgah, Kashan, Iran; 3 Department of Biostatistics and Epidemiology, School of Health, Kashan University of Medical Sciences, Pezeshk Blvd, 5th of Qotbe Ravandi Blvd – Pardis Daneshgah, Kashan, Iran

**Keywords:** Asthma, mHealth, Mobile phone, Self-care

## Abstract

**Background:**

Mobile health (mHealth) has good potential for promoting self-care in patients suffering from chronic diseases. The patients' positive attitude toward this technology is a key factor for the successful implementation. The present study was conducted to investigate the asthma patients' use of mobile phone functionalities and their desire to receive self-care services through this technology.

**Methods:**

This survey study was conducted in Iran in 2018. The study population consisted of 146 patients suffering from asthma. The data collection tool was a questionnaire containing items on the demographic characteristics of patients, current use of mobile phone functionalities, and desire to use them for receiving self-care services. Data were analyzed using descriptive and analytical statistics.

**Results:**

Out of the 160 questionnaires distributed, 146 (91.25%) were completed. The majority of the participants had smartphones (84.9%). Less than half of the participants occasionally used mobile phone functionalities including mobile phone calls (42.5%) and mobile Internet (40.4%) to receive asthma-related information. A significant number of the participants had never used smartphone applications (72.6%) and E-mail (66.4%) to receive asthma-related information. The participants had their greatest use of Internet search, followed by social media, to receive information about asthma symptoms, allergenic and irritating substances, medicinal therapy, and how to use therapy aids. The participants were most willing to use social media for receiving asthma information, communicating with other patients, receiving reminders about doctor's appointment, and receiving warnings about the lack of asthma control.

**Conclusion:**

In Iran as a developing country, asthma patients use Internet search mostly to receive instructional information and are willing to use social media rather than other mobile phone functionalities to receive self-care services. These patients believe that mobile phones are appropriate for receiving instructional information and reminders.

## Background

Asthma is a chronic and common disease of the airways that affects more than 339 million people globally in 2016 [1]. According to WHO estimates, there were 417,918 deaths due to asthma at the global level and 24.8 million disability-adjusted life years (DALYs) attributable to Asthma in 2016 [2, 3]. Despite the extensive use of evidence-based guidelines and available therapy options, symptoms of asthma have not been controlled in patients with asthma effectively [4–6], so that almost 50% of them have symptoms more than once a week [7, 8]. There is much evidence that self-care by patients with asthma have beneficial outcomes, including reduced hospital stays, better pulmonary function, reduced symptoms, and general compliance with treatments [9–11].

One of the approaches for promoting self-care skills in patients with asthma is to provide self-care services (i.e., providing basic information about the nature of asthma, avoiding exposure to allergen and triggers, treating with drugs, providing alerts and reminders to patients, and how to use the therapeutic tools [12]) through information technology tools (e.g. the Internet, mobile phones, and computer software). Given the increasing worldwide use of smartphones, mobile health (mHealth) technologies can work as promising tools to improve self-care in patients with asthma by providing support services such as communication information, providing learning materials, and sending reminders for behavior change [13, 14]. mHealth, as a component of electronic health, is related to the use of mobile phones and other wireless technologies to improve the provision of health-related services. Evidence shows that mHealth tools, including short message service (SMS), applications (apps) and other related technologies, provide a good opportunity for improving patient communications and health care provision [15–20]. Moreover, ease of use, portability and ubiquity in all regions are the potential benefits of mHealth tools for the prevention, diagnosis, treatment and care of diseases and also increasing the access to health services and reducing the costs incurred [21].

There is limited evidence on the efficacy of mHealth in terms of asthma self-care. Two studies showed the positive effect of these interventions on the quality of life, symptoms of disease, and adherence to treatment in patients with asthma [22, 23]. Nevertheless, a study conducted by Ryan et al. (2012) found that using mobile phone apps had no significant effects on asthma self-care outcomes [24]. One of the key factors for successful implementation of mHealth interventions for improving patients’ self-care is considering their attitude toward these technologies [19, 25]. Based on the studies, two studies were conducted in developed countries in this field, both of which showed a positive attitude of patients with asthma toward modern technologies, including mobile phones, SMS, Internet and E-mail, but they also had concerns about the use of these technologies including its time-consuming nature and costs [26, 27]. This indicates the lack of studies in this field and especially its absence in developing countries. Therefore, further studies on the use of mobile phone functionalities by patients with asthma and their desire to use for self-care purposes, especially in developing countries, are necessary.

Given that no similar studies have yet been conducted in developing countries on asthma patients' use of and willingness to use mobile phone functionalities to receive self-care services, the present study was aimed to investigate the patients' current use of mobile phone functionalities (i.e. phone calls, SMS, apps, Internet browsing, E-mail, and social media) to receive self-care services and their willingness to use these functionalities. Understanding these aspects will contribute to the design and implementation of mHealth interventions to help promote self-care in patients with asthma.

## Methods

### Study design, setting and population

This survey study was conducted in Iran in 2018 to investigate asthma patients' use of and desire to use mobile phone functionalities to support self-care. The study population consisted of patients with asthma referred to the only allergy and asthma clinic in Kashan, Iran, with at least one year since the confirmation of their asthma diagnosis and who owned a mobile phone and had at least a high school diploma. In a similar study conducted by Pinnock et al. [26], the mean and standard deviation (SD) of the total score of mobile phone use by patients with asthma was 7.1 ± 2.4, and considering a confidence interval of 95% and the accuracy of 0.4 in estimating the mean score, the minimum required sample size was calculated as 138 patients.

### Questionnaire

To collect data, relevant literature was reviewed and then a questionnaire was designed according to the views expressed by experts about asthma-related information (from asthma and allergy subspecialists) and also about mobile phone functionalities (from health information management and medical informatics experts). To determine the face validity, the questionnaire was first distributed among two health information management and medical informatics experts and its face validity was approved. The content validity of the questionnaire was assessed by 12 experts (in health information management, medical informatics, and allergy and asthma specialization). The validity of each item (i.e. relevance and clarity) plus the validity index of the entire tool (i.e. comprehensiveness) were determined based on a Likert scale (from 1 = ‘unfavorable’ to 4 = ‘totally favorable’). The content validity of each item was assessed based on the content validity index (CVI), and if less than 0.7, the item was revised and modified. The content validity ratio (CVR) was also determined based on Lawshe’s Table [28] and the items with values less than 0.56 were eliminated. The reliability of the questionnaire was determined by the split-half method and its Cronbach's alpha was 0.88.

Following the validation of the instrument, the resultant questionnaire had 38 items in three main sections: demographic information (seven items), overall use of mobile phone functionalities (20 items) and, desire to use mobile phone functionalities (11 items) (Additional file 1). Out of these, five items had "Yes" and "No" answers. A "Yes" answer scored one point and a "No" answer zero points, and six items were four-choice questions, with ‘everyday’ = 1 point, ‘several times per week’ = 0.6, ‘occasionally’ = 0.3 and ‘never’ = 0. The items on the current patients’ use from overall use of mobile phone functionalities (ten items) and the patients’ desire to use (ten items) were scored such that using or the desire to use each functionality were given one point, and no use or no desire to use were given zero points. The sum of the scores from the two sections of the questionnaire (i.e. use of and desire to use) were categorized into three groups as low, medium or high based on 25th and 75th percentiles.

### Data collection

Samples were selected using convenience sampling method [29]. To collect data, the researcher visited the allergy and asthma clinic. Before completing the questionnaire, the eligible patients signed informed consent forms, in which they were briefed on the study objectives and their voluntary participation in the study and the anonymity and confidentiality of their information were ensured; accordingly, their scores were used through coding. Participants completed the self-report questionnaire while waiting to see their physician and any ambiguities in the answers were resolved by asking questions from the respondent. Out of 160 questionnaires distributed among patients with asthma referred to the allergy and asthma clinic, 146 patients participated in the study.

### Statistical analysis

Data were analyzed in Statistical Package for the Social Sciences (SPSS) 22 (IBM SPSS Statistics, IBM Corporation, Armonk, NY, USA). First, the number and frequency percentage of the demographic variables, accessibility and use of mobile phone functionalities were calculated. Then, the mean and standard deviation of the quantitative variables as well as the frequency of the patients' use and desire to use were calculated in terms of the demographic variables. The sum of the scores of the two sections (i.e. use and desire to use) was then calculated. Based on the 25th and 75th percentiles, patients were categorized into three groups in terms of their rate of use: ‘low’ (scores < 12), ‘moderate’ (scores of 12–15.92) and ‘high’ (scores > 15.92). Also, in terms of their desire to use, patients were categorized as ‘low’ (scores < 11), ‘moderate’ (scores of 11–15), and ‘high’ (scores of > 15). The maximum score that could be obtained in two different groups (i.e. use and desire to use) were, respectively, 80 and 71. Frequency percentage was also calculated for each of the items. The relationships between the demographic variables and the questionnaire sections (i.e. use and desire to use) were assessed using the Chi-square or Fisher's exact tests. *P* < 0.05 was taken as the level of statistical significance.

## Results

### Participants' characteristics

In this study, 160 questionnaires were distributed among the participants, of which 146 were willing to complete the questionnaire, with a response rate of 91.25%. Of the 146 participating patients with asthma, 91 (62.3%) were female. Participants' mean age was 34.4 ± 13.3 years. A total of 81 patients (55.5%) had higher than bachelor’s degree and 144 (98.6%) lived in city. A total of 119 patients (81.5%) had asthma for nine years or less. In terms of the severity of asthma, 42.5% of the participants had the "mild persistent" type, 30.1% "moderate persistent", 9.6% "severe persistent" and 4.8% "intermittent" asthma (Table 1).

**Table 1:** Participants' demographic information (N = 146)

Demographic variable	Frequency	Percentage
Gender		
Female	91	62.3
Male	55	37.7
Age (years)		
25 and younger	48	32.9
25–45	67	45.9
45 and above	31	21.2
Mean ± SD^a^	34.4 ± 13.3	
Education level		
Bachelor's degree or less	65	44.5
Higher than bachelor's degree	81	55.5
Place of residence		
City	144	98.6
Village	2	1.4
Duration of asthma		
9 years or less	119	81.5
More than 9 years	27	18.5
Mean ± SD	4.97 ± 4.594	
Severity of asthma^b^		
Mild persistent	62	42.5
Moderate persistent	44	30.1
Severe persistent	14	9.6
Intermittent	7	4.8
Not known	19	13

^**a**^Standard Deviation (SD)

^b^Classified according to the Expert Panel Report 3 (EPR-3): Guidelines for the Diagnosis and Management of Asthma [30]

Table 2 presents the general findings regarding the asthma patients' use and desire to use mobile phone. Out of the 146 participants, 90 (61.6%) used mobile phones to receive asthma care services, 125 (85.6%) had access to the Internet through mobile phone, 124 (84.9%) had smartphones, and 24 (16.4%) had at least one asthma-related app on their mobile phone. Overall, 127 patients (87%) were willing to use mobile phones to receive asthma-care services.

**Table 2:** Overall use of and desire to use mobile phone functionalities by the asthma patients (N = 146)

Items	Number (%)	
	Yes	No
Use of mobile phones to receive asthma care services	90 (61.6)	56 (38.4)
Mobile Internet access	125 (85.6)	21 (14.4)
Have smart mobile phone (ability to install apps)	124 (84.9)	22 (15.1)
Installing mobile apps related to asthma care	24 (16.4)	122 (83.6)
Desire to use mobile phones to receive asthma care services	127 (87)	19 (13)

Table 3 demonstrates the asthma patients' use of mobile phone functionalities to receive asthma-related information in four categories, namely every day, several times per week, occasionally, and never. The results showed that the majority of the patients have never used any mobile phone functionalities to receive asthma-related information; however, they occasionally used mobile phone calls (42.5%) and SMS (36.3%) to receive information and used their mobile Internet (40.4%), social media (39%) and apps (22.6%) to search for and access asthma-related information, and also used E-mail services on their mobile phone to communicate with others (26.7%).

**Table 3:** The frequency of use of mobile phone functionalities by the asthma patients (N = 146)

Items	Everyday (%)	Several times per week (%)	Occasionally^a^ (%)	Never (%)
Receiving asthma-related information through mobile phone calls (to friends, relatives, doctors and nurses)	3 (2)	7 (4.8)	62 (42.5)	74 (50.7)
Receiving asthma-related information through SMS (to friends, relatives, doctors and nurses)	1 (0.7)	7 (4.8)	53 (36.3)	85 (58.2)
Using mobile Internet to search for asthma-related information	1 (0.7)	22 (15.1)	59 (40.4)	64 (43.8)
Using social media (such as Telegram channels) to access asthma-related information	4 (2.7)	18 (12.3)	57 (39)	67 (45.9)
Using mobile E-mail to communicate with others (friends, relatives, doctors and nurses) to receive asthma-related information	0	10 (6.9)	39 (26.7)	97 (66.4)
Using of apps (software) to access asthma-related information	2 (1.4)	5 (3.4)	33 (22.6)	106 (72.6)

^a^About once a week

The results showed that the participants had made their greatest use of the Internet search for the purpose of receiving information about asthma warning symptoms and allergenic and irritating substances (43.2%), medicinal therapy (39.7%) and how to use therapy aids (35.6%). The participants had made their greatest use of social media for receiving reminders about the peak expiratory flow (PEF) test (52.1%), communicating with other patients (34.9%), being warned about the lack of asthma control and being reminded of taking medications on time (32.9%), and being reminded of their vaccination schedule and doctor or nurse appointments (31.5%). Meanwhile, the patients with asthma had a greater desire to use social media (compared to the other functionalities of mobile phones) for receiving information about asthma warning symptoms (54.1%), medicinal therapy (54.1%), allergenic and irritating substances (54.1%) and how to use therapy aids (52.1%). Moreover, 56.8% of the participants were willing to use social media to communicate with other patients, 50.7% to be reminded of their appointments with the doctor or nurse and vaccination schedule, 52.4% to be reminded of taking medications on time, 52.1% to be reminded of taking the PEF test, and 52.7% to be warned about the lack of asthma control (Additional file 2). The results showed that education level, place of residence, severity of asthma, and duration with asthma had no significant relationships with the patients' use of mobile phone functionalities or their views about this technology (Additional file 3).

## Discussion

### Principal findings

The majority of the participants in this study had smartphones and access to the Internet on their mobile phones. Almost half of the participants occasionally used mobile phone calls and mobile Internet to receive asthma-related information. A significant number of the patients had never used mobile phone apps or E-mails to receive asthma-related information. The patients had made their greatest use of mobile phone functionalities through the Internet search, followed by social media, for the purpose of receiving information about asthma warning symptoms and allergenic and irritating substances, medicinal therapy and how to use therapy aids. The participants were most willing to use social media to receive information, communicate with other patients and be reminded of their doctor's appointments, and warned about their lack of asthma control.

The present findings showed that a significant number of the patients had Internet access on their mobile phones, which agrees with the results of other studies on the access of patients with asthma (2017) [31], pregnant women (2014) [32] and African–American families (2014) [33] to the Internet. In contrast, a Pew Research Center report found that Internet access rates among African–Americans were relatively low [34]. Due to recent technological advances, there has been a significant increase in the use of mobile technologies and Internet access. So that areas with low Internet access are also increasing [35]. Therefore, the availability of these tools provides new mechanisms for providing self-care for chronic diseases such as asthma. In addition, the majority of the participants in the present study were willing to use their mobile phones to receive asthma care services. Similarly, Fonseca et al. [36], reported in a study that a large majority of patients were willing and ready to use communication technologies such as cell phones and the web to help them manage their asthma. In another study, the most important reason for patients' desire to use mobile phone technologies was to save time and engaging patients in their disease management [37]. But in general, to successfully introduce new technologies to patients, their concerns must be addressed and patients must be confident about the benefits of using such technologies.

The results showed that less than half of the participants occasionally used mobile phone calls and the Internet to receive asthma-related information. Similarly, Van de Belt et al. (2013) [38] argued that less than half of Dutch people conducted online search for health-related information occasionally before visiting their doctors and after consulting with the doctor. Also, in a study by Calderón et al. (2017) [31], less than half of patients with asthma used the Internet to seek information about asthma. Despite the potential benefits of searching for health information on the Internet, many patients have raised concerns about the negative effects of this searched health information on the Internet. Therefore, the low quality of information available on the Internet can be stated as one of the reasons for patients' low use of Internet health information seeking, which can lead to patients being misinformed, lead to distress, and increase the tendency towards self-diagnosis or self-treatment [39].

The results of one study showed that 77.3% of patients with asthma used E-mails at least once a week, and E-mail was the only platform through which most of them were interested in receiving information and communicating with physicians [40]. However, in the present study, patients' non-use of E-mail communication may be due to their lack of knowledge about using this feature to communicate, lack of an E-mail account, delay in receiving a response, and a lack of sufficient time for physicians to respond. The results of the study conducted by Singh et al. (in New York 2014) [41] concur with the present study findings in terms of the degree of mobile app usage. They reported that although the majority of adolescents or their caregivers were willing to use mobile health apps, only 26% of them had health-related apps installed, and their feedback revealed that the lack of knowledge, poor finances, and privacy concerns were barriers for downloading or using medical apps. Therefore, it seems necessary for physicians to carefully review the information content of the mobile app, before introducing it to their patients.

In the present study, the participants had their greatest use of mobile phone functionalities through the Internet search, followed by social media, to receive asthma-related information, which agrees with the results of other studies [32, 38, 42, 43]. A systematic review study reported that most pregnant women used the Internet as a source of information [44], while in a recent study, patients with asthma preferred SMS over the Internet search and social media, and their reasons for this preference were the low costs and reliability of SMS [31]. In another study, Chisolm et al. [45] analyzed the potential reasons for asthma and diabetes patients' use or lack of use of the Internet, and proposed the lack of time, interest, access and medical necessity as the reasons for not using the Internet, and argued that the reasons for using the Internet were its usefulness, ease of use, medical necessity, and up-to-date nature. In that study, health literacy and demographic factors were significantly linked to the Internet use. Considering that the Internet and social media technologies, in particular, are an important source of health information and the use of online social groups is also increasing, therefore, patients can use these technologies to share experiences and offer support to their peers.

In the present study, the patients were most willing to use social media for the purpose of receiving information, communicating with other patients, being reminded of doctor's appointments, and being warned about their lack of asthma control. Similarly, Ramirez et al. [46] reported that more than 70% of the patients in primary care clinics in Los Angeles were willing to use social media to communicate with other patients with similar problems. Calderón et al. [31] argued that young participants were inclined to use WhatsApp to receive information and be in direct contact with their doctor about asthma. Previous studies have shown how social media, such as Facebook and Twitter, have been effective in improving the outcomes of patients with chronic diseases such as human immunodeficiency virus (HIV), breast cancer and chronic tobacco use [47–49]. These studies demonstrated that social media encourage patients to share health information with others, to teach them about diseases and their treatment, and also to communicate with other patients with similar symptoms who are receiving similar therapies. Nevertheless, privacy concerns are often proposed as the barriers to use social media [50]. Like many emerging technologies, access to information must be controlled to address privacy concerns and safeguards must be put in place to reduce the risk of privacy breaches.

### Strength and limitations

Based on the review of literature, the present study is the first to have been conducted in a developing country on asthma patients' use and desire to use all the functionalities of mobile phones for receiving self-care services. The results can provide a basis for future studies on the design and development of mobile health interventions for patients with asthma. The present study included a sample of 146 patients with asthma in one city, which may be considered as a limitation. Moreover, all the participants were selected from one medical center, since it was the only allergy and asthma clinic in the city of Kashan.

### Implications for practice and future research

Given the evidence found regarding the willingness of patients with asthma to use mobile phone functionalities for receiving self-care services, the following measures are recommended for improving the acceptance of these tools. Clinical professionals should take into account the concerns of these patients about the quality of information provided through mobile phones and also give them assurances about the benefits of using these tools. In the present study, due to the lack of knowledge about asthma self-care apps and the lack of confidence in the informational content of these apps, the patients made little use of them. The cooperation of health care professionals appears necessary for the design, development, and implementation of these apps. The present study determined asthma patients' informational needs and their preferred types of functionalities for receiving asthma self-care services. Designing future interventions based on these informational needs thus appears imperative. Further studies are recommended on these patients' use of and desire to use mobile health technologies in larger populations.

## Conclusion

Currently, patients with asthma often use the Internet search to receive information about asthma symptoms and allergenic and irritating substances, medicinal therapies and how to use therapy aids, and have a more positive attitude toward the use of social media compared to other mobile phone functionalities for receiving these services. Although the present study showed that the Internet and social media are preferred by patients with asthma for self-care, further studies should be conducted to evaluate the quality of asthma-related information in these media.

## Supplementary information

**Additional file 1** English Questionnaire.

**Additional file 2** The frequency of use and desire to use mobile phone functionalities for receiving information, as reminders and warnings in asthma patients (N=146).

**Additional file 3** The frequency of use and desire to use mobile phone functionalities in asthma patients in terms of demographic variables (N=146).

## Data Availability

The datasets used and/or analysed during the current study available from the corresponding author on reasonable request.

## Abbreviations

mHealth: Mobile Health
US: United States
SMS: Short message service
Apps: Applications
UK: United Kingdom
SD: Standard deviation
CVI: Content validity index
CVR: Content validity ratio
SPSS: Statistical Package for the Social Sciences
PEF: Peak expiratory flow
HIV: Human immunodeficiency virus
