# Supplementary material for: The use of mobile phone functionalities by patients with asthma and their desire to use for self-care purposes
Source: BMC Med Inform Decis Mak. 2020 Oct 30;20:281. doi: 10.1186/s12911-020-01301-z (PMC7602318; doi:10.1186/s12911-020-01301-z)
Supplement: Supplementary file 1 — Additional file 1 English Questionnaire. [file 12911_2020_1301_MOESM1_ESM.docx]

**Additional file 1: English Questionnaire**

**Demographic characteristics**

Year of Birth: ……………….

Gender:  Female  Male

Place of residence:  City  Village

Job: ……………….......

Level of education:  Associate's degree  Bachelor's degree  Masters' degree  Ph.D.

Duration of asthma: …………….… (Year)

Severity of asthma: Mild persistent Moderate persistent severe persistent intermittent

1. Do you use a mobile phone to receive asthma care services?  Yes  No
2. How often do you use mobile phone calls (to friends, relatives, doctors and nurses) to receive asthma-related information?

Everyday  Several Times per Week  Occasionally  Never

1. How often do you use SMS (to friends, relatives, doctors and nurses) to receive asthma-related information?

Everyday  Several Times per Week  Occasionally  Never

1. Do you have internet access through your mobile phone?  Yes  No
2. How often do you use your mobile internet to search for asthma-related information?

Everyday  Several Times per Week  Occasionally  Never

1. How often do you use social media (such as Telegram channels) to access asthma-related information?

Everyday  Several Times per Week  Occasionally  Never

1. How often do you use mobile email to communicate with others (friends, relatives, doctors and nurses) to receive asthma-related information?

Everyday  Several Times per Week  Occasionally  Never

1. Do you have a smartphone (iPhone/Android etc.)?  Yes  No
2. Do you have asthma related apps on your mobile phone?  Yes  No
3. How often do you use mobile apps (software) to access asthma-related information?

Everyday  Several Times per Week  Occasionally  Never

* Which mobile phone functionality do you currently use to receive asthma care services (from a doctor or nurse)? (You can also select several options). Other items please be mentioned here. …………………...

| Asthma care services | | Mobile phone functionalities | | | | | | | |
| --- | --- | --- | --- | --- | --- | --- | --- | --- | --- |
|  |  | None | Phone/Voice Call | SMS | Internet Search | Social Media | Email | Software/Apps | Video Call |
| 11 | Receiving information about asthma warning symptoms (cough, wheezing, shortness of breath) |  |  |  |  |  |  |  |  |
| 12 | Receiving information about medicinal therapy |  |  |  |  |  |  |  |  |
| 13 | Receiving information about allergenic and irritating substances (air pollution) |  |  |  |  |  |  |  |  |
| 14 | Receiving information about how to use therapy aids (PEF test) |  |  |  |  |  |  |  |  |
| 15 | Communicating with other patients |  |  |  |  |  |  |  |  |
| 16 | Reminders about doctor or nurse appointments |  |  |  |  |  |  |  |  |
| 17 | Reminders for influenza vaccination |  |  |  |  |  |  |  |  |
| 18 | Reminders for medication use |  |  |  |  |  |  |  |  |
| 19 | Reminders for PEF test |  |  |  |  |  |  |  |  |
| 20 | Warning about lack of asthma control |  |  |  |  |  |  |  |  |

21. Would you like to use mobile phone to receive asthma care services? Yes  No the reason for your unwillingness.........................

* Which mobile phone functionality would you like to receive asthma care services (from a doctor or nurse)? (You can also select several options). Other items please be mentioned here. …………………...

| Asthma care services | | Mobile phone functionalities | | | | | | | |
| --- | --- | --- | --- | --- | --- | --- | --- | --- | --- |
|  |  | None | Phone/Voice Call | SMS | Internet Search | Social Media | Email | Software/Apps | Video Call |
| 22 | Receiving information about asthma warning symptoms (cough, wheezing, shortness of breath) |  |  |  |  |  |  |  |  |
| 23 | Receiving information about medicinal therapy |  |  |  |  |  |  |  |  |
| 24 | Receiving information about allergenic and irritating substances (air pollution) |  |  |  |  |  |  |  |  |
| 25 | Receiving information about how to use therapy aids (PEF test) |  |  |  |  |  |  |  |  |
| 26 | Communicating with other patients |  |  |  |  |  |  |  |  |
| 27 | Reminders about doctor or nurse appointments |  |  |  |  |  |  |  |  |
| 28 | Reminders for influenza vaccination |  |  |  |  |  |  |  |  |
| 29 | Reminders for medication use |  |  |  |  |  |  |  |  |
| 30 | Reminders for PEF test |  |  |  |  |  |  |  |  |
| 31 | Warning about lack of asthma control |  |  |  |  |  |  |  |  |
