# Supplementary material for: The use of mobile phone functionalities by patients with asthma and their desire to use for self-care purposes
Source: BMC Med Inform Decis Mak. 2020 Oct 30;20:281. doi: 10.1186/s12911-020-01301-z (PMC7602318; doi:10.1186/s12911-020-01301-z)
Supplement: Supplementary file 2 — Additional file 2 The frequency of use and desire to use mobile phone functionalities for receiving information, as reminders and warnings in asthma patients (N=146). [file 12911_2020_1301_MOESM2_ESM.docx]

**Additional file 2: The frequency of use and desire to use mobile phone functionalities for receiving information, as reminders and warnings in asthma patients (N=146)**

| Items | | None | Phone/Voice Call | SMS | Internet Search | Social Media | Email | Software/Apps | Video Call |
| --- | --- | --- | --- | --- | --- | --- | --- | --- | --- |
| Use (%) | Receiving information about asthma warning symptoms (cough, wheezing, shortness of breath) | 62 (42.5) | 15 (10.3) | 4 (2.7) | 63 (43.2) | 32 (21.9) | 0 | 2 (1.4) | 1 (0.7) |
|  | Receiving information about medicinal therapy | 65 (44.5) | 16 (11) | 2 (1.4) | 58 (39.7) | 33 (22.6) | 0 | 3 (2.1) | 0 |
|  | Receiving information about allergenic and irritating substances (air pollution) | 60 (41.1) | 6 (4.1) | 3 (2.1) | 63 (43.2) | 42 (28.8) | 0 | 2 (1.4) | 0 |
|  | Receiving information about how to use therapy aids (PEF test) | 70 (47.9) | 8 (5.5) | 5 (3.4) | 52 (35.6) | 30 (20.5) | 0 | 3 (2.1) | 0 |
|  | Communicating with other patients | 81 (55.5) | 15 (10.3) | 6 (4.1) | * | 51 (34.9) | 0 | 2 (1.4) | 2 (1.4) |
|  | Reminders about doctor or nurse appointments | 77 (52.7) | 22 (15.1) | 6 (4.1) | * | 46 (31.5) | 0 | 2 (1.4) | 0 |
|  | Influenza vaccination reminder | 85 (58.2) | 14 (9.6) | 5 (3.4) | * | 46 (31.5) | 0 | 2 (1.4) | 0 |
|  | Medication use reminder | 80 (54.8) | 15 (10.3) | 6 (4.1) | * | 48 (32.9) | 0 | 4 (2.7) | 0 |
|  | Reminders for PEF test | 21 (14.4) | 29 (19.9) | 37 (25.3) | * | 76 (52.1) | 1 (.7) | 9 (6.2) | 1 (0.7) |
|  | Warning about lack of asthma control | 86 (58.9) | 11 (7.5) | 4 (2.7) | * | 48 (32.9) | 0 | 1 (0.7) | 0 |
| Desire (%) | Receiving information about asthma warning symptoms (cough, wheezing, shortness of breath) | 20 (13.7) | 20 (13.7) | 35 (24) | 32 (21.9) | 79 (54.1) | 4 (2.7) | 10 (6.8) | 1 (0.7) |
|  | Receiving information about medicinal therapy | 18 (12.3) | 24 (16.4) | 33 (22.6) | 31 (21.2) | 79 (54.1) | 3 (2.1) | 10 (6.8) | 1 (0.7) |
|  | Receiving information about allergenic and irritating substances (Air pollution) | 21 (14.4) | 19 (13) | 32 (21.9) | 33 (22.6) | 79 (54.1) | 2 (1.4) | 9 (6.2) | 1 (0.7) |
|  | Receiving information about how to use therapy aids (PEF test) | 21 (14.4) | 21 (14.4) | 29 (19.9) | 29 (19.9) | 76 (52.1) | 3 (2.1) | 9 (6.2) | 1 (0.7) |
|  | Communicating with other patients | 33 (22.6) | 23 (15.8) | 24 (16.4) | * | 83 (56.8) | 3 (2.1) | 5 (3.4) | 2 (1.4) |
|  | Reminders about doctor or nurse appointments | 23 (15.8) | 36 (24.7) | 38 (26) | * | 74 (50.7) | 2 (1.4) | 6 (4.1) | 1 (0.7) |
|  | Influenza vaccination reminder | 25 (17.1) | 29 (19.9) | 39 (26.7) | * | 74 (50.7) | 1 (0.7) | 6 (4.1) | 1 (0.7) |
|  | Medication use reminder | 22 (15.2) | 30 (20.7) | 36 (24.8) | * | 76 (52.4) | 1 (0.7) | 7 (4.8) | 1 (0.7) |
|  | Reminders for PEF test | 26 (17.8) | 27 (18.5) | 38 (26) | * | 76 (52.4) | 1 (0.7) | 7 (4.8) | 1 (0.7) |
|  | Warning about lack of asthma control | 28 (19.2) | 25 (17.1) | 36 (24.7) | * | 77 (52.7) | 1 (0.7) | 6 (4.1) | 1 (0.7) |

*** Not Applicable**
