# Supplementary material for: The use of mobile phone functionalities by patients with asthma and their desire to use for self-care purposes
Source: BMC Med Inform Decis Mak. 2020 Oct 30;20:281. doi: 10.1186/s12911-020-01301-z (PMC7602318; doi:10.1186/s12911-020-01301-z)
Supplement: Supplementary file 3 — Additional file 3 The frequency of use and desire to use mobile phone functionalities in asthma patients in terms of demographic variables (N=146). [file 12911_2020_1301_MOESM3_ESM.docx]

**Additional file 3: The frequency of use and desire to use mobile phone functionalities in asthma patients in terms of demographic variables (N=146)**

| Demographic Variable | | Use (%) | | | | Desire (%) | | | |
| --- | --- | --- | --- | --- | --- | --- | --- | --- | --- |
|  |  | Low | Moderate | High | P-value | Low | Moderate | High | P-value |
| Gender | Female | 33 (36.3) | 35 (38.5) | 23 (25.3) | 0.965 | 62 (68.1) | 8 (8.8) | 21 (23.1) | 0.716 |
|  | Male | 21 (38.2) | 21 (38.2) | 13 (23.6) |  | 37 (67.3) | 7 (12.7) | 11 (20) |  |
| Age (years) | 25 and younger | 17 (35.4) | 20 (41.7) | 11 (22.9) | 0.394 | 31 (64.6) | 5 (10.4) | 12 (25) | 0.763 |
|  | 25 to 45 | 21 (31.3) | 27 (40.3) | 19 (28.4) |  | 44 (65.7) | 8 (11.9) | 15 (22.4) |  |
|  | 45 and above | 16 (51.6) | 9 (29) | 6 (19.4) |  | 24 (77.4) | 2 (6.5) | 5 (16.1) |  |
| Education | Less than bachelor’s degree | 29 (44.6) | 19 (29.2) | 17 (26.2) | 0.106 | 49 (75.4) | 6 (9.2) | 10 (15.4) | 0.183 |
|  | Higher than bachelor’s degree | 25 (30.9) | 37 (45.7) | 19 (23.5) |  | 50 (61.7) | 9 (11.1) | 22 (27.2) |  |
| Place of residence | City | 53 (36.8) | 56 (38.9) | 35 (24.3) | 0.497 | 98 (68.1) | 14 (9.7) | 32 (22.2) | 0.161 |
|  | Village | 1 (50) | 0 | 1 (50) |  | 1 (50) | 1 (50) | 0 |  |
| Severity of Asthma | Mild | 21 (33.9) | 24 (38.7) | 17 (27.4) | 0.781 | 41 (66.1) | 9 (14.5) | 12 (19.4) | 0.606 |
|  | Moderate | 16 (36.4) | 19 (43.2) | 9 (20.5) |  | 31 (70.5) | 2 (4.5) | 11 (25) |  |
|  | Severe | 6 (42.9) | 3 (21.4) | 5 (35.7) |  | 8 (57.1) | 1 (7.1) | 5 (35.7) |  |
|  | Intermittent | 2 (28.6) | 4 (57.1) | 1 (14.3) |  | 4 (57.1) | 1 (14.3) | 2 (28.6) |  |
|  | Not known | 9 (47.4) | 6 (31.6) | 4 (21.1) |  | 15 (78.9) | 2 (10.5) | 2 (10.5) |  |
| Duration of asthma | 9 years and less | 40 (33.6) | 47 (39.5) | 32 (26.9) | 0.174 | 78 (65.5) | 12 (10.1) | 29 (24.4) | 0.321 |
|  | 10 years and longer | 14 (51.9) | 9 (33.3) | 4 (14.8) |  | 21 (77.8) | 3 (11.1) | 3 (11.1) |  |
